# The Impact of PPE Availability on Moral Distress Among EMTs During the COVID‐19 Pandemic and Strategies: A Review and a Logic Model

**DOI:** 10.1002/hsr2.71130

**Published:** 2025-07-27

**Authors:** Mohadeseh Motamed‐Jahromi, Elsa Vitale, Hamid Torabian, Mohammad Parvaresh‐Masoud

**Affiliations:** ^1^ Department of Public Health, School of Health Fasa University of Medical Sciences Fasa Iran; ^2^ Directorate of Health and Nursing Professions ASL Bari Bari Italy; ^3^ Department of Pediatric Nursing, School of Nursing Qom University of Medical Sciences Qom Iran; ^4^ Department of Emergency Medicine, Paramedical Faculty Qom University of Medical Sciences Qom Iran

**Keywords:** emergency medical technicians, logic model, morals, personal protective equipment

## Abstract

**Background and Aims:**

The COVID‐19 pandemic has placed an unprecedented strain on healthcare systems worldwide, with frontline healthcare workers, including Emergency Medical Technicians (EMTs), facing numerous challenges. One of the most significant issues has been the availability and adequacy of personal protective equipment (PPE). This study explores the relationship between PPE availability and moral distress among EMTs during the COVID‐19 pandemic and proposes a logic model to address it.

**Methods:**

In this narrative review, a goal‐based search was conducted in several databases, and related articles were selected, and the desired relationships and strategies were extracted from them. Finally, a logic model was presented.

**Results:**

Inadequate PPE availability on EMTs can lead to heightened moral distress, psychological strain, and ethical dilemmas, affecting their well‐being and the quality of care they provide. To mitigate moral distress effectively, strategies such as ensuring a reliable PPE supply chain, efficient distribution systems, prioritizing PPE allocation, establishing clear protocols, promoting mental health support, and ethical decision‐making are crucial. The logic model is drawn by establishing relationships between inputs, activities, outputs, and outcomes of the program.

**Conclusion:**

The availability of PPE is critical to the health and safety of EMTs during emergencies, especially pandemics. The proposed logic model can guide stakeholders to work together, address PPE availability and moral distress among EMTs, and improve well‐being and quality of care.

## 1 Introduction

The COVID‐19 pandemic has placed an unprecedented strain on healthcare systems worldwide, with frontline workers, including Emergency Medical Technicians (EMTs), facing numerous challenges [1]. One of the most significant issues has been the availability and adequacy of personal protective equipment (PPE) [2]. The lack of proper PPE has not only increased the risk of infection among EMTs but has also contributed to heightened levels of moral distress [3, 4].

Moral distress occurs when healthcare professionals are unable to carry out what they believe to be the ethically appropriate course of action due to institutional constraints or conflicting obligations [5]. EMTs play a crucial role in the healthcare system, providing pre‐hospital care and transportation to patients, including those with suspected or confirmed COVID‐19 [1]. The nature of their work requires close contact with patients, often in uncontrolled environments, making the use of PPE essential for their safety and the safety of their patients.

The global shortage of PPE at the beginning of the pandemic forced many EMTs to work with inadequate protection, leading to increased anxiety, fear, and moral distress [1, 6]. This shortage not only posed a risk to the health and safety of EMTs but also presented ethical dilemmas as they strived to provide optimal care while facing the challenges of inadequate protection. Studies have highlighted the profound effects of moral distress on the mental health and well‐being of healthcare workers, with increased risks of burnout, depression, and posttraumatic stress disorder (PTSD) among those exposed to high levels of moral distress [3, 4].

The search results highlight a gap in research regarding the relationship between access to PPE and moral distress among EMTs during the COVID‐19 pandemic. To address this gap, a narrative review was conducted to investigate the impact of PPE access on EMTs' moral distress and to propose strategies based on existing literature. Finally, a logic model is drawn to provide a comprehensive understanding of the challenges facing EMTs, practical solutions, and outcomes.

## 2 Methods

The narrative review method is used for data collection in this study, which is based on a comprehensive literature search conducted using various computerized databases such as PubMed, Scopus, Science Direct, and Google Scholar. The search covered articles published before April 6, 2024, with no limitations on the date of studies or type of articles. The study language was only English, and eight keywords were selected after consulting with emergency nursing and ethics experts. The keywords were as follows: PPE, Prehospital Care Emergency Medical Services (EMS), COVID‐19 pandemic, moral distress, ethical dilemmas, PPE availability, strategies, and implications.

### 2.1 Inclusion and Exclusion Criteria

The search results provided include articles that focus on PPE availability, moral distress among EMTs during the COVID‐19 pandemic, and strategies to address these issues. Articles without full text or those whose full text was not available were excluded.

### 2.2 Data Extraction and Analysis

**Figure 1:**
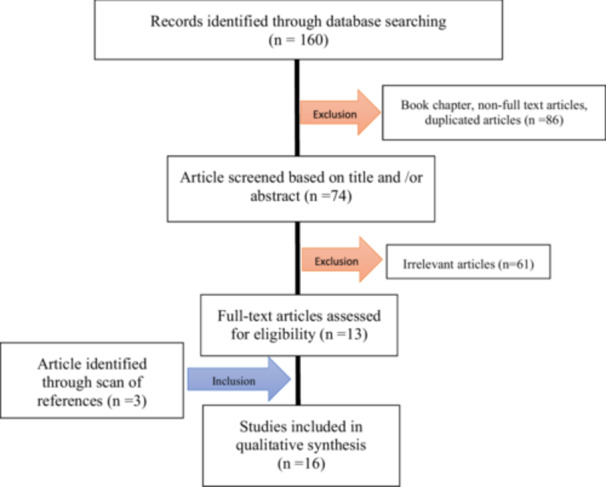
Search process and study identification.

After the final selection of articles, information was extracted from the full text. Among the 160 sources retrieved, 16 sources were selected based on their complete relevance to the objectives of the study. The flow diagram for this review is shown in Figure 1.

### 2.3 Logic Model

A logic model is a visual representation of a program or intervention that specifies the relationships between the program's inputs, activities, outputs, outcomes, and impacts [7]. It is a tool used to plan, implement, and evaluate programs, helping to clarify program goals, identify key stakeholders, and ensure that all components are aligned to achieve desired outcomes [8]. At the end of the article, we proposed a logic model for addressing moral distress among EMTs during the COVID‐19 pandemic.

## 3 Results

### 3.1 Importance of PPE Availability

The importance of PPE availability is paramount, especially in the context of Prehospital Care Emergency Medical Services (EMS) and the COVID‐19 pandemic. Adequate access to PPE is crucial for reducing occupational risks and protecting the health and safety of EMTs who are at the forefront of responding to medical emergencies and infectious disease outbreaks [9]. Ensuring the availability of appropriate PPE is essential for minimizing the risk of exposure to pathogens and defending against various infectious agents encountered during EMS encounters [10, 11]. Studies have shown that the lack of full PPE utilization in EMS encounters, particularly during potential COVID‐19 exposures, can lead to increased moral distress among responders and compromise their well‐being [10]. Proper PPE availability not only safeguards the health of EMS providers but also plays a vital role in preventing the unnecessary depletion of already limited PPE supplies, thereby ensuring continuity in providing essential care [12]. PPE Availability is important from two aspects.

#### 3.1.1 Health and Safety

Adequate PPE availability protects EMTs from exposure to infectious diseases like COVID‐19, ensuring their health and safety [1].

#### 3.1.2 Ethical Dilemmas

When EMTs face a PPE shortage, they often feel compelled to provide care despite the heightened risks to their health. This situation does not imply that they will abandon their patients; rather, they grapple with the ethical implications of delivering care under unsafe conditions [13]. The dilemma arises from their commitment to patient welfare, which clashes with their concerns about safety and well‐being. As a result, EMTs may experience moral distress as they navigate this complex landscape, struggling to reconcile their professional obligations with the reality of inadequate protection. Ultimately, the lack of PPE forces EMTs to confront challenging ethical decisions that can compromise both their safety and the quality of care they strive to deliver [14].

### 3.2 Impact of PPE Availability on Moral Distress

The lack of adequate PPE during the COVID‐19 pandemic had a profound impact on the moral distress experienced by EMTs. EMTs are frontline healthcare providers who are responsible for responding to medical emergencies and transporting patients to hospitals. At the beginning of the pandemic, they faced unprecedented challenges due to the highly contagious nature of the virus and the shortage of PPE, which put them at increased risk of infection. Limited access to PPE on EMTs can have significant implications.

#### 3.2.1 Psychological Strain

The lack of adequate PPE can result in heightened psychological strain among EMTs, leading to increased anxiety, fear, and emotional burden. This can have a profound impact on their mental well‐being, potentially contributing to stress and other mental health challenges [6].

#### 3.2.2 Ethical Concerns

EMTs may encounter ethical dilemmas when faced with the challenge of balancing their duty to care for patients with concerns about personal safety due to insufficient PPE. This ethical conflict can create moral distress, as EMTs navigate the complexities of providing optimal care while safeguarding themselves from potential harm [1, 15].

### 3.3 Strategies to Mitigate Moral Distress

**Figure 2:**
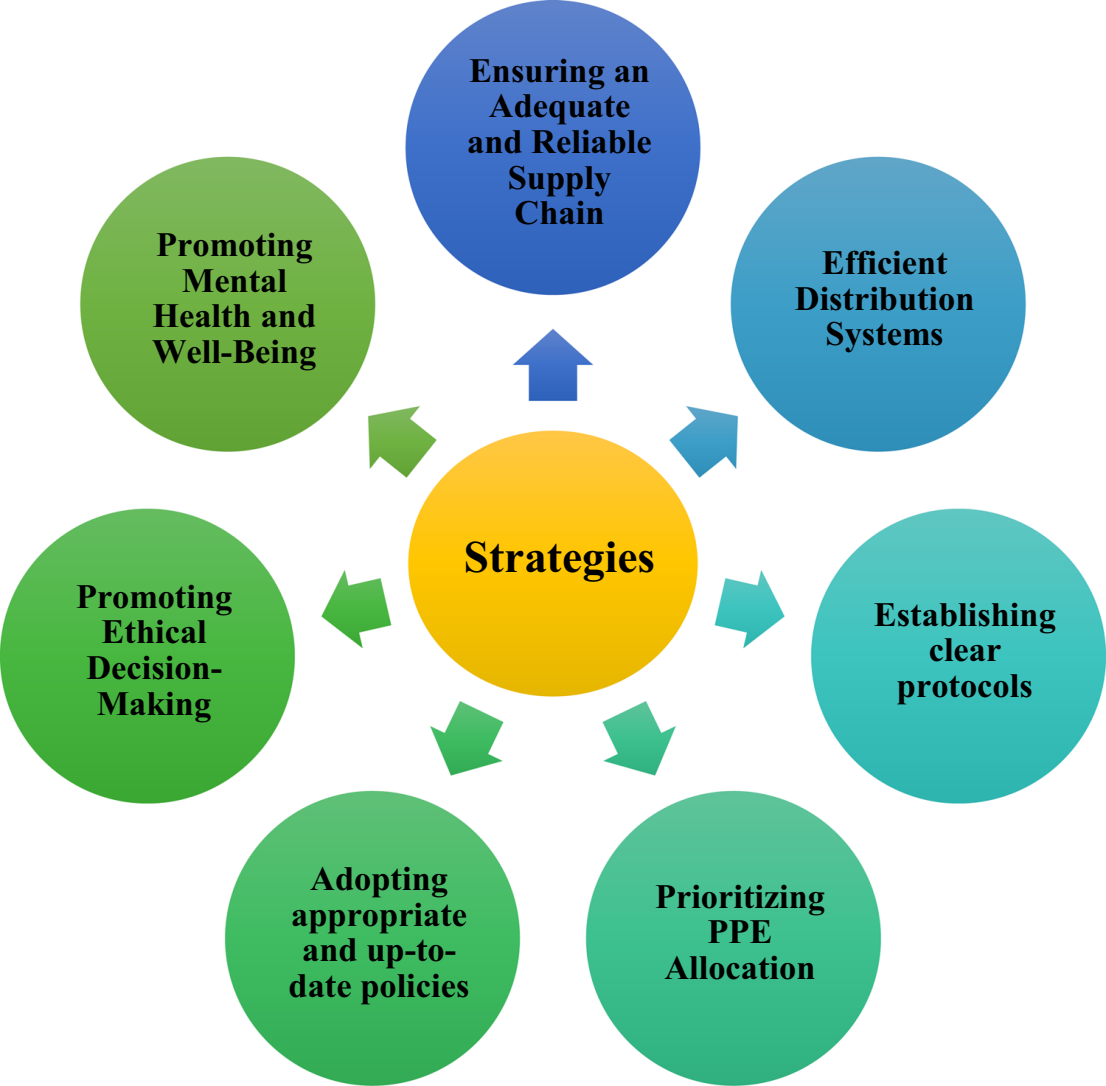
Strategies to reduce moral distress among EMTs during a pandemic.

Several strategies have been identified in the literature and employed by EMS agencies to mitigate moral distress among EMTs during the pandemic. Figure 2 shows strategies for reducing moral distress among EMTs during the COVID‐19 pandemic.

#### 3.3.1 Ensuring an Adequate and Reliable Supply Chain

The most effective way to reduce moral distress among EMTs is to ensure they have access to adequate PPE. This includes an N95 mask, gloves, gown, and eye protection. Governments, healthcare institutions, and supply chain managers should work together to establish a consistent and reliable supply of PPE to meet the needs of EMTs and frontline healthcare workers [16, 17]. EMTs also play a vital role in advocating for their needs within their organizations. By articulating their experiences and the challenges they face due to PPE shortages, EMTs can raise awareness among leadership about the critical importance of adequate supplies. Engaging in discussions regarding resource allocation and contributing to feedback mechanisms can significantly influence decision‐making processes [18]. Ultimately, while systemic issues contribute to PPE shortages, frontline workers can still engage in advocacy and collaboration to improve their working conditions and ensure they have the necessary tools to provide safe care.

#### 3.3.2 Efficient Distribution Systems

Efficient distribution systems must be in place to ensure that PPE is available when and where EMTs need it. PPE should be provided free of charge to EMTs because they should not bear the financial burden of protecting themselves and their patients [19, 20].

#### 3.3.3 Prioritizing PPE Allocation

To effectively prioritize PPE allocation, a systematic approach is essential, particularly for high‐risk areas like Prehospital Care Emergency Medical Services (EMS), ensuring that EMTs have timely access to necessary PPE when responding to medical emergencies and infectious disease outbreaks [21]. However, this focus may inadvertently lead to shortages for other healthcare workers, potentially compromising their safety and ability to provide care. To address these ethical challenges, a transparent and equitable allocation framework is needed, prioritizing PPE distribution based on exposure risk, the critical nature of the work, and the overall impact on patient care [13]. Involving diverse stakeholders in decision‐making will ensure a balanced approach that respects the rights and safety of all healthcare workers while providing necessary protection to those at greatest risk [22]. Such collaboration can help mitigate moral distress within the healthcare system and promote a culture of shared responsibility during crises like the COVID‐19 pandemic.

#### 3.3.4 Establishing Clear Protocols

EMTs need clear protocols for donning and doffing PPE, as well as for managing patients with suspected or confirmed COVID‐19. These protocols should be based on the latest scientific evidence and should be updated regularly as new information becomes available [23].

#### 3.3.5 Promoting Mental Health and Well‐Being

EMS agencies need to prioritize the mental health and well‐being of their EMTs. This includes providing access to counseling, peer support, and other interventions. EMS agencies should also create a culture of resilience and support, where EMTs feel comfortable talking about their experiences and seeking help when needed [24, 25].

#### 3.3.6 Promoting Ethical Decision‐Making

EMTs should be guided and supported in navigating ethical dilemmas related to providing care in the face of PPE shortages and emphasize the importance of following ethical principles while ensuring personal and patient safety [26]. To navigate these challenges effectively, it is essential to support EMTs with structured ethical frameworks [27]. Incorporating the Ethical Infection Prevention and Control (EIPAC) Decision‐Making Framework, developed by the Association for Professionals in Infection Control and Epidemiology (APIC) and IPAC Canada, can significantly enhance the ethical decision‐making process for EMTs during PPE shortages. This framework provides a systematic approach to identify relevant facts, values, and principles while exploring various options available to EMTs [28]. By applying the EIPAC framework, EMTs can make informed decisions that prioritize both patient care and personal safety. This framework encourages collaboration among healthcare professionals, helping to address moral tensions that arise in challenging situations [29]. It emphasizes the importance of ethical considerations guiding actions, ensuring that EMTs uphold their commitment to providing safe and effective care.

#### 3.3.7 Adopting Appropriate and Up‐to‐Date Policies

Governments should develop and implement policies that ensure that EMTs have access to adequate PPE and mental health support. These policies should be based on the latest scientific evidence and should be updated regularly as new information becomes available. EMS agencies should adopt policies and procedures that promote EMTs' mental health and well‐being. These policies should include provisions for providing access to counseling, peer support, and other interventions [30, 31].

## 4 A Proposed Logic Model

In the context of the COVID‐19 pandemic and its impact on EMTs, a logic model could be used to address PPE availability and moral distress among EMTs.

**Figure 3:**
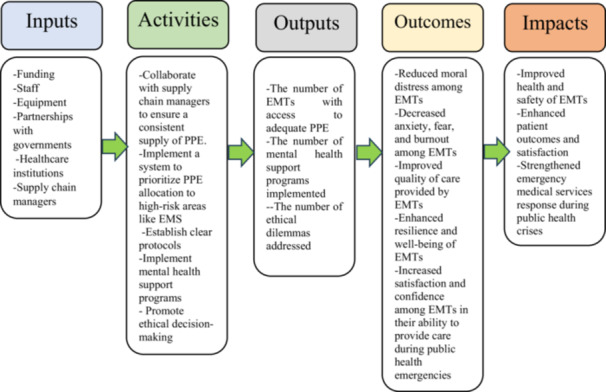
A proposed logic model for addressing moral distress among EMTs during the COVID‐19 pandemic.

A logic model is a tool used to summarize a program and its intended outcomes, which can be used to communicate with stakeholders. It outlines the relationships between the program's inputs, activities, outputs, and outcomes. The inputs of the model are the resources required to implement the program [32], including funding, staff, equipment, and partnerships with governments, healthcare institutions, and supply chain managers. Activities are the actions taken to achieve the program's goals [33], including ensuring an adequate and reliable supply chain, prioritizing PPE allocation, establishing clear protocols, implementing mental health support programs, and promoting ethical decision‐making. Outputs are the direct products or services produced by the program [34], including the number of EMTs with access to adequate PPE, the number of mental health support programs implemented, and the number of ethical dilemmas addressed. Outcomes are the short‐term and intermediate‐term effects of the program [35], including reduced moral distress among EMTs, improved mental health and well‐being, and improved quality of care delivered to patients. Impacts are the long‐term effects of the program [34], including improved health and safety for EMTs and better outcomes for patients. Figure 3 shows a proposed logic model for addressing moral distress among EMTs during the COVID‐19 pandemic.

This logic model proposed by this study allows stakeholders to better understand the relationships between the various components of the program and ensure that all efforts are coordinated to achieve the desired results. It can also help identify areas for improvement and inform policy decisions, ultimately leading to reduced moral distress for EMTs and safer outcomes for the educators who serve them.

## 5 Limitations of the Study

This review has several limitations, including a focus on English literature, which may exclude relevant studies in other languages, and the narrative review methodology, which can introduce selection bias. Furthermore, the scope of identified strategies may not encompass all potential interventions, and the focus on EMTs may overlook the experiences of other healthcare workers facing similar challenges. In addition, the scarcity of relevant literature and the absence of established flowcharts specific to this topic presented significant challenges in constructing a comprehensive and well‐supported logic model. This limitation underscores the need for further research in this area.

## 6 Conclusion

The availability of PPE is paramount in safeguarding the health and safety of EMTs, especially during crises like the COVID‐19 pandemic. The impact of inadequate PPE availability on EMTs can lead to heightened moral distress, psychological strain, and ethical dilemmas, affecting their well‐being and the quality of care they provide. Strategies such as ensuring a reliable PPE supply chain, efficient distribution systems, prioritizing PPE allocation, establishing clear protocols, promoting mental health support, and ethical decision‐making are crucial to mitigate moral distress effectively. By implementing these strategies and utilizing a logical model to guide efforts, stakeholders can work collaboratively to address PPE availability and moral distress among EMTs. This coordinated approach aims to reduce moral distress, improve mental well‐being, enhance patient care quality, and ultimately ensure the safety and effectiveness of frontline healthcare providers. Prioritizing PPE availability and addressing moral distress not only protects EMTs but also contributes to a resilient and sustainable healthcare system capable of responding effectively to future challenges.

## 7 Implications for Practice

The study highlights the need for enhanced PPE supply chain management to ensure consistent access for EMTs, thereby reducing moral distress. Implementing ethical decision‐making frameworks, such as the EIPAC framework, can guide EMTs in navigating ethical dilemmas related to PPE shortages. Mental health support should be prioritized to address the psychological impacts of moral distress. Interdisciplinary collaboration among healthcare professionals can foster a supportive environment for discussing ethical concerns. Finally, ongoing research is necessary to explore the long‐term effects of PPE shortages and evaluate the effectiveness of implemented strategies in real‐world settings.

## Author Contributions

**Mohadeseh Motamed‐Jahromi:** conceptualization, methodology, software, data curation, formal analysis, validation, investigation, writing – original draft, writing–review and editing, visualization, resources. **Elsa Vitale:** conceptualization, methodology, data curation, investigation, writing – original draft, writing–review and editing, resources, visualization. **Hamid Torabian:** conceptualization, writing –original draft, writing – review and editing, visualization, methodology, resources. **Mohammad Parvaresh‐Masoud:** conceptualization, methodology, software, data curation, supervision, resources, formal analysis, validation, investigation, writing – original draft, writing – review and editing, visualization, funding acquisition, project administration.

## Conflicts of Interest

The authors declare no conflicts of interest.

## Transparency Statement

The lead author, Mohammad Parvaresh‐Masoud, affirms that this manuscript is an honest, accurate, and transparent account of the study being reported; that no important aspects of the study have been omitted; and that any discrepancies from the study as planned (and, if relevant, registered) have been explained.

## Data Availability

The data that support the findings of this study are available from the corresponding author upon reasonable request.
